# Glucose variability and mood in adults with diabetes: A systematic review

**DOI:** 10.1002/edm2.152

**Published:** 2020-07-14

**Authors:** Linda T. Muijs, Caterina Racca, Maartje de Wit, Annelies Brouwer, Thomas H. Wieringa, Ralph de Vries, Erik H. Serné, Daniël H. van Raalte, Femke Rutters, Frank J. Snoek

**Affiliations:** ^1^ Medical Psychology Amsterdam UMC Vrije Universiteit Amsterdam Amsterdam The Netherlands; ^2^ Internal Medicine Diabetes Center Amsterdam UMC Vrije Universiteit Amsterdam Amsterdam The Netherlands; ^3^ Epidemiology and Biostatistics Amsterdam UMC Vrije Universiteit Amsterdam Amsterdam The Netherlands; ^4^ Medical Library Amsterdam UMC Vrije Universiteit Amsterdam Amsterdam The Netherlands

**Keywords:** adult, affect/mood, blood glucose variability, diabetes mellitus, systematic review

## Abstract

**Aims:**

To systematically review the literature regarding the association between glucose variability (GV) and mood in adults with diabetes, appraise the used methods and make suggestions for future research.

**Methods:**

A systematic review of literature published up to May 2019 was performed. Abstracts and full texts were screened independently in duplicate. Experimental and observational studies reporting the association between GV and mood in adults with type 1 diabetes or type 2 diabetes were evaluated. A descriptive analysis of the extracted data was conducted, along with a quality assessment.

**Results:**

Out of the 2.316 studies screened, eight studies met our criteria. Studies used a variety of measures and metrics to determine GV and mood. Four studies used continuous glucose monitoring (CGM). An association between GV and mood was found in four studies when correlating either postprandial glucose rate of increase with current mood or multiday GV with mood measured retrospectively. The other four studies did not find any association.

**Conclusions:**

There is no clear empirical support for a link between GV and mood in adults with type 1 and type 2 diabetes. More rigorous research is warranted using CGM and ecological momentary assessment of mood to assess if and under what conditions an association between GV and mood exists.

## Graphical abstract

The literature regarding the association between glucose variability (GV) and mood in adults with diabetes is systematically reviewed. Methods used are summarized and evaluated, and suggestions for future research and clinical implications are made.

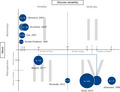

**Novelty statement**

1 **What is already known**

- There is a long‐standing interest in the association between glucose variability (GV) and mood in persons with diabetes.
- Empirical evidence regarding this association has not been systematically reviewed.

2 **What this study has found**

- Four of the eight included studies used continuous glucose monitoring (CGM).
- A significant association was found between a higher rate of postprandial glucose increase and more negative mood symptoms.
- No other evident patterns between GV and mood emerged.
- Higher quality experimental and observational studies are needed using CGM and ecological momentary assessment.

3 **What are the clinical implications of the study**

- Increasing use of sensor technology in routine care will increase insight into glucose over time.
- Digital mood diaries can identify individual patterns over time and review outcomes with significant others and professionals

## 1 INTRODUCTION

The association between mood and glucose variability (GV) in persons with diabetes has been a topic of interest since the 1930s.^1^ Stress and negative mood have been assumed to explain unpredictable and extreme blood glucose fluctuations often referred to as “brittle diabetes”.^2^ In the early 1980s, the attention shifted to the opposite direction, that is the effect of “diabetic instability” on psychological problems.^3^

To date, when investigating the association between different static glucose levels on mood, experimental research in healthy volunteers showed no consistent effect,^4^ while some studies in persons with diabetes suggest that both hyperglycaemia and hypoglycaemia can induce negative mood states, including anxiety, sadness and agitation.^5^, ^6^, ^7^ Also, self‐monitoring of glucose values can elicit strong emotional responses, often negative and related to a sense of failure.^8^ When investigating the link between the dynamics in glucose levels and mood, blinded continuous glucose monitoring (CGM) technology provides the opportunity to observe the association between GV and mood, as noted by Rausch et al a decade ago.^9^

It is important to note that some metrics of GV strongly correlate with mean glucose.^10^ However, the relationship between mean glucose and mood does not capture the daily emotional impact of glucose excursions. A better understanding of this association might help to reduce the uncertainty around the interrelationship between one's blood glucose level and mood, which has been identified as one of the most frequently endorsed problem areas by both people with type 1 and type 2 diabetes.^11^ Moreover, new diabetes medications and diabetes technologies can help to achieve less glucose variability and more “time in range”.^12^ With increasing uptake of CGM use in research, a literature overview can help to enhance our understanding of the potential psychological benefits of improved glucose stability for persons with diabetes.

In this systematic review, we aim to give an overview of the existing literature regarding the association between GV and mood in adults with diabetes mellitus. Furthermore, we discuss the strengths and weaknesses of the methods used to examine this association and make suggestions for future research.

## 2 METHODS

### 2.1 Data sources and searches

A literature search was performed based on the Preferred Reporting Items for Systematic Reviews and Meta‐Analyses (PRISMA) statement.^13^ PubMed, EMBASE and PsycINFO (EBSCO) databases were searched using the following terms (including synonyms and closely related words) as index terms or free‐text words: “Diabetes Mellitus”, “Blood glucose variability” and “Mood” to identify studies that examined the association between glucose variability and mood in adults with type 1 and type 2 diabetes (for full search, see Appendix S1). References of included studies and relevant reviews were checked for additional relevant articles. The initial search was performed in July 2018 and updated in May 2019. Covidence software was used to manage the screening process.^14^

### 2.2 Study results and study selection

**Figure 1:**
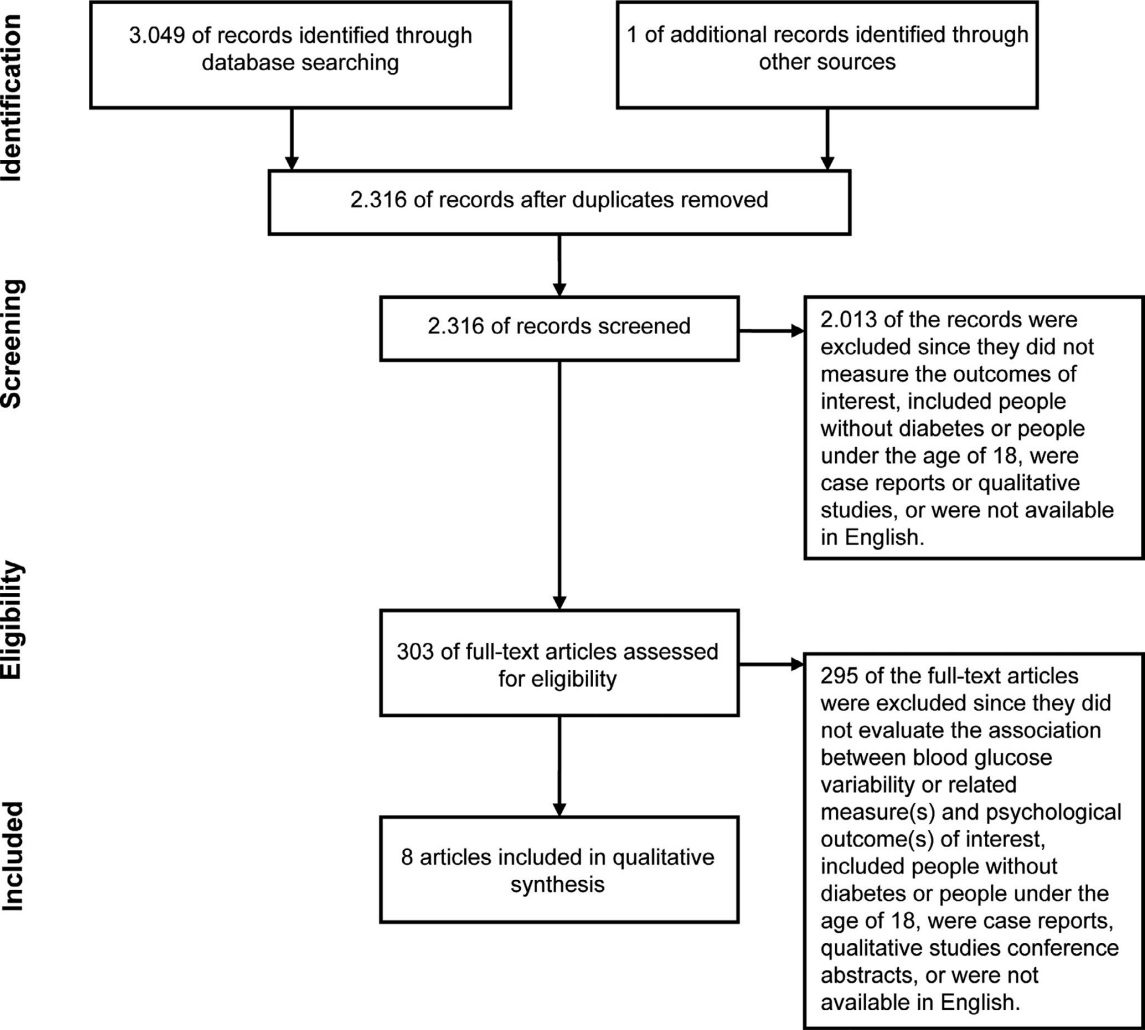
Flow diagram of study selection

The literature search generated a total of 3.049 references: 944 in PubMed, 1.786 in Embase.com and 319 in PsycINFO. After removing duplicates of references that were selected from more than one database, 2.316 references remained. The flow chart of the search and selection process is presented in Figure 1.

Peer‐reviewed studies published in English that examined the association between glucose variability and mood in adults with diabetes were included. Reviews, conference abstracts, qualitative studies, editorials and case report forms were excluded. Inclusion criteria were as follows: observational or experimental research designs; assessing glucose variability (eg “fluctuation”, “instability” and “glucose rate of change”); and assessment of mood (eg emotion, well‐being and affect) (see Appendix S2 for detailed inclusion criteria).

Study titles and abstracts were screened, and subsequently, full texts were reviewed for inclusion in duplicate by seven reviewers (CR, LTM, MdW, FJS, THW, FR and AB) independently. Three reviewers (CR, LTM and MdW) discussed conflicts until agreement was reached.

### 2.3 Data extraction and quality assessment

Data were extracted independently and in duplicate by three reviewers (CR, LTM and MdW) including study design, country of participants recruited, study duration, (demographical and diabetes‐related) participant characteristics, indices of GV and mood, metrics used, statistical test used to examine the association between the outcomes of interest, and results. The method for measuring GV was identified as either continuous glucose monitoring (CGM) or self‐monitoring of blood glucose (SMBG), and indices of GV were reported according to the metrics described by Siegelaar et al^15^ or as reported by the author. The time frame was operationalized as the duration and frequency of the glucose values measured. Self‐reported mood was classified as measured either retrospectively (eg “Over the past weeks/hours, I felt irritable”) or momentarily (eg “At the moment, I’m feeling irritable”). In addition, we noted the instrument used to assess mood and extracted the time(s) of measurement within the study period. The quality of the studies was rated independently and in duplicate by five reviewers (CR, LTM, MdW, FR and AB), using the NIH Quality Assessment Tool suited for both observational cohort and cross‐sectional studies.^16^ Discrepancies in quality rating were discussed (CR, LTM, M.d.W) until consensus was reached. In line with Mikkelsen et al,^17^ the quality of the studies was rated as poor (≤7), fair (8‐11) or good (≥12), based on a summary score with a minimum of 0 and a maximum of 14.

### 2.4 Data synthesis

Given the heterogeneity of measurements and study designs, a standard meta‐analysis was not feasible. We therefore conducted a descriptive analysis of the collected data. For data synthesis, we grouped the studies using a two‐dimensional map based on the time frame of the measurement of GV (within 1 day, ie intraday, vs. more than 1 day, ie multiday, on the *X*‐axis) and mood (momentary vs. retrospective on the *Y*‐axis), resulting in four quadrants.

## 3 RESULTS

### 3.1 Study characteristics

A flowchart of the study selection process is shown in Figure 1. Eight studies were included with a total of 1.200 participants, ranging from 14 to 976 participants per study, including people with type 1 diabetes (n = 4)^18^, ^19^, ^20^, ^21^ and type 2 diabetes (n = 4).^22^, ^23^, ^24^, ^25^ Five studies were conducted in North America^20^, ^22^, ^23^, ^24^, ^25^ and three in Europe.^18^, ^19^, ^21^ Research designs were observational (n = 5),^18^, ^19^, ^24^, ^25^, ^26^ crossover randomized controlled trials (n = 2)^21^, ^22^ and experimental (n = 1).^20^ Details of the study design and participant characteristics are shown in Table 1.

**Table 1:** Design and participant characteristics of studies that evaluated the association between glucose variability and mood

Study ID	Study characteristics						Participant characteristics				
	Primary or secondary research question	Design	Study duration	Country	Sample size	Females (%)	Age in years (mean ± SD; range)	Type of diabetes	Diabetes duration in years (mean ± SD; range)	Treatment regimen (if insulin, CSII/MDI)	HbA1c in mmol/mol; % (mean ± SD (range))
Ahola 2018	Secondary	Observational	NA	Finland	976	59	48 ± 14; 36‐60	1	NR	Insulin, NR	64 ± NR (56‐73); 8.0 ± NR (7.3‐8.8)
Cox 2007	Secondary	Crossover RCT	24 weeks (2× 12‐week treatment period)	USA	28	NR	NR (of 45 randomly assigned 52.6 ± 11.9)	2	NR (of 45 randomly assigned 11.9 ± 7.5)	Insulin + oral agent (metformin)	NR
Gonder‐Frederick 1990	Secondary	Experimental	1 day + 1 day follow‐up (12 weeks later)	USA	14	71	38.5 ± 13.2; 22‐65	1	16 ± 1.1; 1‐39	Insulin, NR	NR; 10.9 ± 3.2 (6.6‐17.0)
Hermanns 007	Primary	Observational	Differs across participants: 48.8 h (mean)	Germany	36	22	31.1 ± 10; NR	1	14.7 ± 7.1; NR	Insulin, 0 (25.7%) CSII; 26 (74.3%) MDI	NR; 8.4 ± 1.8 (NR)
Johansson 1999	Secondary	Crossover RCT	8 weeks (2× 4 weeks + 4‐week wash‐out period)	Sweden	37	59	46.6 ± 12.4; 22‐69	1	26.0 ± 15; 4.0‐53.0	Insulin, NR	NR; 7.9 ± 1.0 (6.1‐10.4)
Kovachev 2003	Primary	Observational	3 to 4 weeks	USA	36	58	50 ± 11; NR	2	10 ± 9; NR	38% insulin (other NR), NR	NR
Penckofer 2012	Primary	Observational	72 h	USA	23	100	Median 51 (40‐67)	2	10 (2‐26)	NR	Median 8.0% (6.0‐13.0)
Wagner 2017	Primary	Observational	7 days	USA	50	74	58.8 ± 11.9	2	NR	Insulin use: 57%	8.3% ± 1.5%

Abbreviations:

RCT, randomized control trial; NA, not applicable; NR, not reported; USA, the United States of America.

### 3.2 Quality assessment of included studies

Using the NIH Quality Assessment Tool, the quality of seven studies^18^, ^19^, ^20^, ^21^, ^22^, ^23^, ^25^ was judged to be fair, and one study^24^ was judged to be of poor quality (see Table 2). Appendix S3 gives an overview of the quality assessment of each study.

**Table 2:** Results of studies that evaluated the association between glucose variability and mood

Study ID	Operationalization of outcomes of interest						Evaluated association between outcomes of interest		
	Glucose variability			Mood			Statistical analysis	Results (effect sizes)	Quality assessment
	Method (CGM/SMBG)	Metrics	Time frame	Method (retrospective/momentary)	Instrument	Time of measurement			
Ahola 2018	SMBG	SD	2× 3 days usual care within 2 to 3 months	Retrospective (over the past week): online/paper questionnaire	Beck Depression Inventory	NR	Generalized linear regression (*B*)	0.40*	Fair
Cox 2007	SMBG	BGRATE	Before breakfast and dinner until 1 h later	Momentary: HHC	9 mood symptoms in clusters: (a) depressive, (b) anxious, (c) energetic	(1)1 h after breakfast (2)1 h after dinner	Pearson's correlation (*r*)	Group lispro mixture (1)/(2) (a) 0.45**/0.56** (b) 0.43*/0.49** (c) 0.11/ −0.16 Group glargine (1)/(2) (a) 0.39*/0.53** (b) 0.41*/0.51** (c) 0.13/ −0.60	Fair
Gonder‐Frederick 1990	CGM^a^	Absolute and signed (positive/negative) prestressor‐poststressor BG changes	Prestressor (prior to 20‐min stress condition) until poststressor (40 min after completion of stress condition) on day 1 and day 2 (12 weeks later)	Momentary: paper checklist	Mood states: frustrated, happy, anxious/tense, angry, glad, frightened	Prestressor and poststressor condition on day 1 and day 2 (12 weeks later) (prestressor‐poststressor mood change)	Spearman's correlation (*r*)	NR/ NS	Fair
Hermanns 2007	CGM	(A) Absolute change in BG (B) CV	60 min prior to mood rating, within on average 48.8‐h period	Momentary: HHC	UWIST Mood Adjective Checklist: (a) tension, (b) hedonic tone, (c) anger, (d) energetic arousal	Several times (mean = 15.7 ± 8.4 times) during valid CGM period	Multilevel regression analyses (*z*‐scores)	(A) (a) −0.50 (b) 0.82 (c) −0.72 (d) −0.07 (B) (a) −0.55 (b) −0.40 (c) 0.36 (d) −0.08	Fair
Johansson 1999	SMBG	SD	Every two days 5 times a day (before breakfast, lunch and dinner, 90 min after dinner and before bedtime), for 4 weeks per study period (cisapride, placebo)	Retrospective (over the past weeks): online/paper questionnaire	Well‐being questionnaire: (a) depressed mood, (b) anxiety, (c) energy, (d) positive well‐being, (e) general well‐being	At baseline, after 4, 8 and 12 weeks	Pearson's correlation (*r*)	Group Cisapride: (a) 0.40* (b) 0.26 (c) −0.52** (d) −0.32* (e) −0.42* Group Placebo (a) 0.38* (b) 0.26 (c) −0.40* (d) −0.19 (e) −0.34*	Fair
Kovachev 2003	SMBG	BGRI	Postprandial consecutive BG changes between 0, 1, 2 and 3 h	Momentary: HHC	6 mood symptoms (a) nervous/anxious, (b) irritable/frustrated, (c) restless/jittery, (d) sad/blue, (e) giddy/funny, (f) don't care/apathetic (g) average magnitude of mood symptoms	Immediately before an SMBG measurement	Correlation (*r*)	After 1 h/2 h/3 h (a) 0.69**/0.48**/0.43* (b) 0.56**/ 0.28/ 0.33 (c) 0.50**/0.52**/0.45* (d) 0.68**/0.53**/0.44* (e) 0.50**/0.07/−0.06 (f) 0.66**/0.54**/0.37 (g) 0.70**/0.49**/0.44*	Fair
Penckofer 2012	CGM	(1) SD (2) CONGA1‐CONGA6 (3)(i) CGM 24‐h average “energy”; (ii) “energy” 1‐12 cycles/24 h	The last full 24‐h CGM record, taken from the protocol period of 72 h	Retrospective (over the past week): online/paper questionnaire	(a) CES‐D: (i) score; (ii) nondepressed (score < 16) vs depressed (score ≥ 16), (b) State Anxiety Inventory (c) State Anger Inventory	At first visit, during 1‐h CGM calibration	Two‐sample t test: (1)/(2)/(3i)/(3ii) and (a) Pearson's correlation (r): (1)/(2)/(3i)/(3ii) and (a)/(b)/(c)	NR/ NS Except: (3ii)(a) 0.54**	Poor
Wagner 2017	CGM	SD	(1) SD of 7 days (2) SD during 10 h following IVR windows (10 AM‐8 PM; 10 PM‐8 AM)	Retrospective (over 10 to 14 h prior to the 10‐h GV measurement): IVR	Positive affect (PA) (enthusiastic, happy, calm, and relaxed) and negative affect (NA) (nervous, mad, sad, and bored) composites: (a) mean PA (b) mean NA (c) SD PA (d) SD NA	Twice daily: 8‐10 AM 8‐10 PM	Pearson's correlation (r) (1) Multilevel regression analyses (*B*) (2)	(1)(a) −0.11 (1)(b) 0.12 (1)(c) −0.14 (1)(d) 0.04 (2)(a) 2.35 (2)(b) −3.10	Fair

Abbreviations:

BGRATE, blood glucose rate of change; BGRI, blood glucose rate of increase; CES‐D, Center for Epidemiological Studies Depression Scale; CGM, continuous blood glucose monitor; CONGA(n), continuous overall net glycaemic action, calculated at n*‐*hour intervals; CV, coefficient of variation; HHC, hand‐held computer; IVR, interactive voice response; NA: not applicable; NR: not reported; NS: not significant; SD, standard deviation; SMBG, self‐monitoring of blood glucose.

^a^ Glucose/insulin infusion system providing continuous BG measurement.

* *P‐*value ≤ .05;

** *P‐*value ≤ .01.

### 3.3 Glucose variability measures

Three studies used the CGM device, called Medtronic MiniMed (CGMS; Medtronic MiniMed), which they blinded for the study participants and allowed only the retrospective analysis of glucose values,^19^, ^24^, ^25^ and four studies used SMBG^18^, ^21^, ^22^, ^23^ to determine GV, using various metrics and time windows as indicators of GV. One study used a glucose/insulin infusion procedure allowing for continuous measurement of blood glucose similar to CGM.^20^ Five studies measured intraday GV,^19^, ^20^, ^22^, ^23^, ^24^ two studies measured GV over multiple days,^18^, ^21^ and one study captured intraday GV as well as over one week.^25^ The GV metrics used as described by Siegelaar et al^15^ were the standard deviation (SD), with time windows ranging from 10 hours up to four weeks,^18^, ^21^, ^24^, ^25^ coefficient of variation (CV)^19^ and continuous overall net glycaemic action (CONGA).^24^ Other GV metrics reported by the authors were intraday change in blood glucose to indicate GV, such as blood glucose rate of change (BGRATE),^19^, ^20^, ^22^, ^23^ and a newly introduced measure called CGM “energy”.^24^

### 3.4 Mood measures

All studies measured negative mood, such as depressive symptoms, anxiety and anger either retrospectively^18^, ^21^, ^24^, ^25^ or momentarily, using subjective mood ratings.^19^, ^20^, ^22^, ^23^ Three studies also assessed positive mood states, for example, “happy” and “hedonic tone”.^19^, ^20^, ^25^ Feeling “energetic” was captured by two studies.^19^, ^22^

### 3.5 Outcomes of association between glucose variability and mood

**Figure 2:**
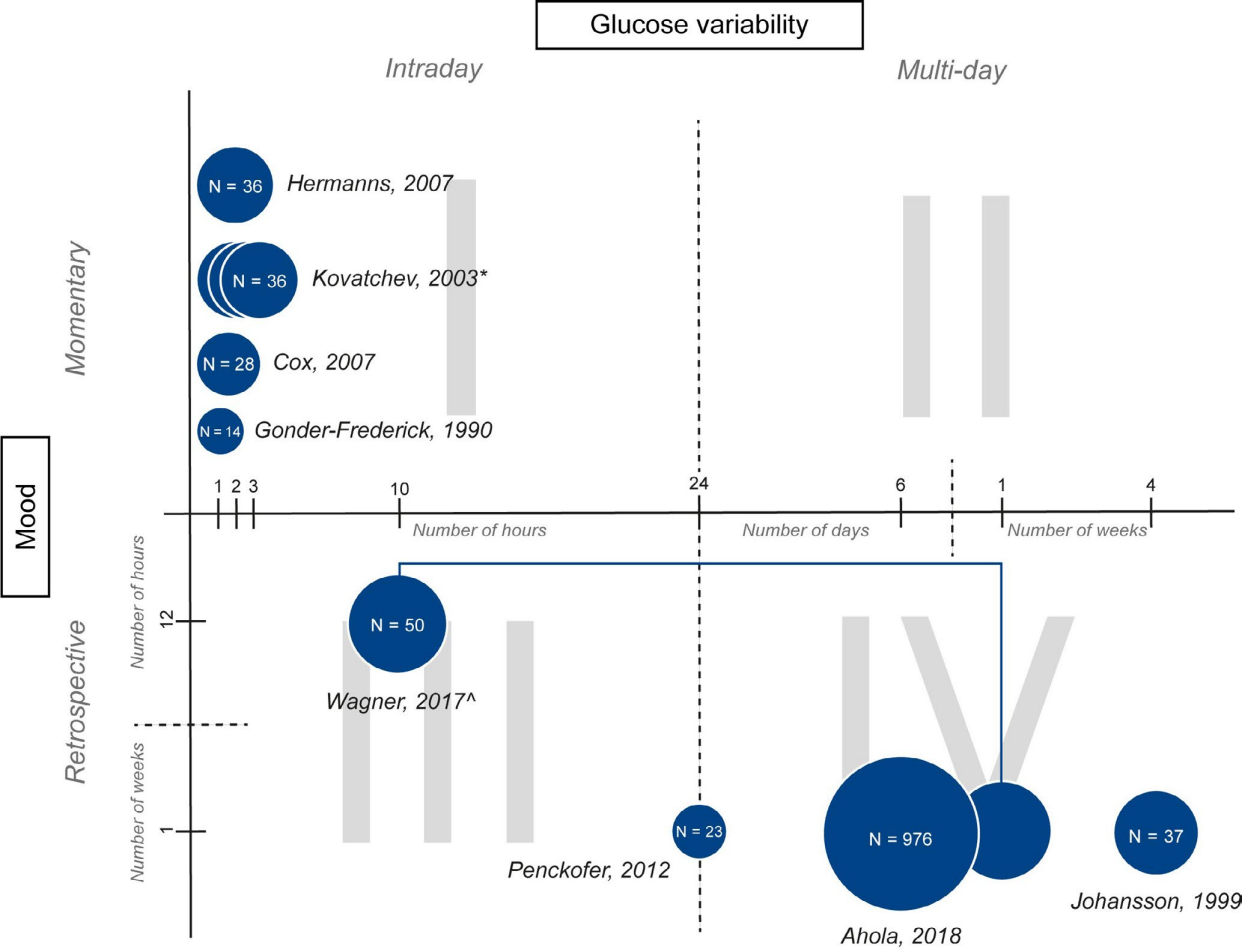
Overview of time frames used to measure glucose variability and mood with sample size representation per study. Footnote: QI: intraday GV and momentary mood; QII: multiday GV and momentary mood; QIII: intraday GV and retrospective mood; QIV: multiday GV and retrospective mood. *Kovatchev^23^ measures GV 1, 2 and 3 hours postmeal. ^Wagner^25^ measures both intraday GV and multiday GV

The association between GV and mood was examined in three ways, as presented in Figure 2. First, the association between *intraday GV and momentary mood* was assessed in five studies^19^, ^20^, ^22^, ^23^, ^24^ (Figure 2, QI). Cox et al^22^ and Kovatchev et al^23^ observed that a higher rate of increase in postprandial glucose values significantly correlated with higher postprandial negative mood symptoms in persons with type 2 diabetes. This association was observed at one hour postmeal^22^ and proved strongest at one hour postmeal.^23^ No correlation was observed between postprandial blood glucose rate of changes and positive (energetic) mood symptoms.^22^ Gonder‐Frederick et al^20^ explored the effect of an active stressor (challenging mental test) on the association between mood and glucose changes in persons with type 1 diabetes. They did not find a significant association between pre‐ to poststressor glucose and mood changes. Hermanns et al^19^ measured intraday GV and momentary mood within a total study period of 48.8 hours on average, in persons with type 1 diabetes. No significant association was observed between mood and GV, measured with CV, or absolute glucose changes, in the 60 minutes prior to the mood rating. Penckofer et al^24^ did not find a significant association between GV, measured with CGM, and state anxiety or state anger in persons with type 2 diabetes.

Second, the association between *intraday GV and retrospective mood* was measured in two studies^24^, ^25^ (Figure 2, QIII). Again, Penckofer et al^24^ did not observe a significant association between GV and depressive symptoms in persons with type 2 diabetes. Wagner et al^25^ determined the correlation between positive or negative mood and GV, measured with the CV, of the 10‐hour period following the mood rating, and found no significant association.

Third, three studies^18^, ^21^, ^25^ assessed the association between *multiday GV and mood measured retrospectively* (Figure 2, QIV). Ahola et al^18^ and Johansson et al^21^ found that higher GV, measured with SMBG, was significantly associated with higher depressive symptom scores in persons with type 1 diabetes. Lower GV was found to be significantly associated with higher positive well‐being scores.^21^ Wagner et al^25^ did not observe any association between GV, measured in a 7‐day period using CGM, and the average positive and average negative mood within the same 7‐day period. Details of the results of the eight studies that evaluated the association between GV and mood are shown in Table 2.

## 4 DISCUSSION

Overall, the results of this systematic review do not provide clear evidence for a link between intraday GV and mood states in adults with diabetes.^19^, ^20^, ^24^, ^25^ A significant association was found between a higher rate of postprandial glucose increase and more negative mood symptoms in persons with type 2 diabetes,^22^, ^23^ warranting further research. The remaining studies seem to suggest a possible cumulative effect of multiday GV on depressive mood assessed retrospectively in adults with type 1 diabetes.^18^, ^21^

Several methodological shortcomings limit the internal validity of the results of the reviewed studies. First, half of the reviewed studies used SMBG to determine GV.^18^, ^21^, ^22^, ^23^ SMBG is less informative than CGM and likely to capture less GV with a lower frequency of self‐testing. Also, SMBG measurements are generally not performed in a blinded manner and the person's appraisal and emotional response to their current blood glucose value may have an impact on their mood.^8^ To test a direct effect of GV on mood, studies would ideally exclude the possibility of a feedback loop, requiring blinding of the glucose test results, which can only be done in a research setting. Continuous glucose monitoring (CGM) offers the best possible opportunity for precise and blinded GV measurement in relation to subjective mood ratings in real life.^9^ However, this would still require the person with diabetes to intermittently ascertain his or her blood glucose control unless glucose control is fully automated. This would, however, prevent the occurrence of extreme glucose excursions that maybe necessary to induce mood changes.^27^

Second, with regard to measuring mood, half of the studies used retrospective questionnaires,^18^, ^21^, ^24^, ^25^ which are more prone to recall bias than momentary assessments,^28^ and are less sensitive to mood fluctuations within one day. Preferably, mood is assessed in a time window close to the measurement of GV, that is fluctuations in glucose values that occur throughout a reasonable time period of interest.^15^ However, the optimal time period between the tested GV window and the correlated current mood rating, or change in mood rating in the previously mentioned GV window, has yet to be determined. It would seem that studies linking GV and momentary mood ratings should at least cover multiple days in total, in order to catch a sufficient and realistic amount of variability in both parameters.

Third, the study populations of six studies were relatively small (less than N = 40),^19^, ^20^, ^21^, ^22^, ^23^, ^24^ while two studies included a selected group of participants^19^, ^25^ limiting external validity. For example, Hermanns et al^19^ studied people with relatively well‐controlled type 1 diabetes admitted to a tertiary clinic, with 75% of CGM time spent within the euglycaemic range. Also, some studies were conducted in persons with type 2 diabetes^22^, ^23^, ^24^, ^25^ with probably less pronounced GV than in type 1 diabetes.^29^ The impact of GV on mood can be assumed to be a function of experiencing extreme glucose excursions, that is amplitude and the frequency of oscillations. Research in type 1 diabetes has established profound effects of severe hypoglycaemia on mood states that may persist over time.^7^, ^30^, ^31^ Similarly, acute hyperglycaemia might alter mood in type 2 diabetes, only above a certain glycaemic threshold.^32^, ^33^

As to the direction of the relationship between GV and mood, almost all studies examined whether GV was a predictor of subsequent mood changes, but reversed causality cannot be excluded. Wagner et al^25^ indeed assessed whether mood was a predictor of subsequent GV, but found no evidence for this direction. It is important to note that none of the reviewed studies used time‐series statistical analysis to model the relationship between GV and mood using temporal data. As suggested by Rausch,^9^ this approach is necessary to accurately determine the direction of the relationship between glucose variability and mood.

More work needs to be done to understand potential mechanisms underlying an association between GV and mood. Gonder‐Frederick et al^20^ found that stress impacted both glucose levels and mood, but did not find support for stress as a mediator of the association between GV and mood. Likewise, Wagner et al^25^ did not find evidence that GV and mood were mediated by diabetes self‐care behaviours. While larger studies are needed and can provide robust data, aggregating findings on a group level may mask interindividual differences in emotional responses to GV. For example, high mood variability can be expected in persons characterized by impulsivity,^34^ while depression is characterized by low mood variability.^35^ Persons can also differ in terms of interoceptive (bodily) awareness, including impaired hypoglycaemia awareness. Other possible moderators of the link between GV and mood include trait anxiety^36^ and sleep^37^, ^38^ that are associated with instable glucose levels as well as poor emotional well‐being.

Another phenomenon that could hypothetically alter the relationship between GV and mood is impaired cardiovascular autonomic modulation,^39^, ^40^ as is the case in cardiovascular autonomic neuropathy, one of the complications of diabetes.^41^ Understanding interindividual differences in emotional reactivity to GV could help predict which persons with diabetes could profit most from more stable blood glucose levels in terms of their emotional health.

To further improve the quality of research in this field, standardization of both GV and mood measures is essential. With the increasing use of CGM, international consensus on clinical targets has recently been established,^12^ with a strong focus on “time in range” as a measure of glycaemic control. It is yet unclear if, how and for whom more time in range translates into improved psychological health, including mood and cognitive functioning. Next to standardization of GV measures,^9^, ^42^ a consensus on the measurement of mood in the context of GV is called for, with focus on ecological momentary assessment (EMA) technology, and grounded in theory of psychological well‐being.^43^ To better understand the dynamics of emotions in the context of GV, the so‐called circumplex model of affect would appear as useful theoretical framework.^44^ This two‐dimensional model allows for assessment of psychological responses combining arousal (activation) and valence (pleasantness). Wagner et al^25^ indeed assessed four of these states in their study, and further research using the circumplex model is warranted.

The strengths of this review are that studies were systematically selected from three databases, and reviews were hand‐searched for more relevant literature. Both observational and experimental studies were included, providing relevant information regarding the research question. The limitations are that no grey literature was searched, non‐English articles were excluded, and experts within the field were not contacted, making it prone to have a biased set of studies.

In conclusion, based on this systematic review of eight studies no firm conclusions can be drawn with regard to the association between GV and mood in adults with type 1 and type 2 diabetes. More and higher quality experimental and observational studies with larger populations over a longer period of time are needed. New technologies, such as blinded CGM and EMA mobile applications, are promising to assess this association more precisely, addressing a question that is perceived to be of high importance from the perspective of persons with diabetes.

## CONFLICT OF INTEREST

No conflicts of interest.

## AUTHOR CONTRIBUTION

LTM, CR, MdW, AB, THW, EHS, DHvR, FR and FJS were involved in the conception and design of the review. LTM, CR, MdW, FJS and RdV conducted the search strategy, and RdV executed the search in three databases. LTM, CR, MdW, FJS, AB, THW and FR carried out the study selection. LTM, CR, MdW, AB and FR done the quality assessment. LTM, CR and MdW extracted data and discussed and resolved conflicts in the selection and extraction process. All authors (LTM, CR, MdW, AB, THW, RdV, EHS, DHvR, FR and FJS) were involved in drafting the manuscript and revising it critically, read and approved the final manuscript and agreed to be accountable for all aspects of the work in ensuring that questions related to the accuracy or integrity of any part of the work are appropriately investigated and resolved.

## ETHICS STATEMENT

Since this review summarizes and informs already published studies, ethical approval is not applicable.

## Supporting information

Supplementaryfile S1‐S3

## Data Availability

Supporting data about the search details, the inclusion and exclusion criteria for full‐text selection and the quality assessment are provided as supplementary information. Other data that support the findings of this study are available from the corresponding author upon reasonable request.
