# Supplementary material for: Glucose variability and mood in adults with diabetes: A systematic review
Source: Endocrinol Diabetes Metab. 2020 Jul 14;4(1):e00152. doi: 10.1002/edm2.152 (PMC7831227; doi:10.1002/edm2.152)
Supplement: Supplementary file 1 — Supplementaryfile S1‐S3 [file EDM2-4-e00152-s001.docx]

# Supplementary File 1: Search terms data bases

## Initial search terms June 2018

PubMed Session Results (29 June 2018)

| Search | Query | Items found |
| --- | --- | --- |
| #6 | #5 NOT ("Animals"[Mesh] NOT "Humans"[Mesh]) | 869 |
| #5 | #4 NOT (("Adolescent"[Mesh] OR "Child"[Mesh] OR "Infant"[Mesh]) NOT ("Adult"[Mesh])) | 916 |
| #4 | #1 AND #2 AND #3 | 1,027 |
| #3 | "Psychology"[Mesh] OR "Emotions"[Mesh] OR emotion*[tiab] OR affect[ti] OR affect[ot] OR mood*[tiab] OR feelings[tiab] OR well-being[tiab] OR wellbeing[tiab] OR depressive symptom*[tiab] | 558,216 |
| #2 | "Diabetes Mellitus"[Mesh] OR diabetes[tiab] OR diabetic*[tiab] OR dm2[tiab] OR niddm[tiab] OR dm 2[tiab] OR t2d*[tiab] OR dm type 2[tiab] OR dm type II[tiab] OR dm1[tiab] OR iddm[tiab] OR dm 1[tiab] OR t1d*[tiab] OR dm type 1[tiab] OR dm type I[tiab] OR mody[tiab] OR lada[tiab] OR midd[tiab] | 612,848 |
| #1 | (blood glucose[tiab] AND mood*[tiab]) OR glucose rate of change*[tiab] OR blood sugar varia*[tiab] OR glucose varia*[tiab] OR glycemic varia*[tiab] OR glycaemic varia*[tiab] OR ((glycemia[tiab] OR glycaemia[tiab]) AND (varia*[tiab])) OR hypoglycem*[tiab] OR hypoglycaem*[tiab] OR hypo-glycem*[tiab] OR hypo-glycaem*[tiab] OR hyperglycem*[tiab] OR hyperglycaem*[tiab] OR hyper-glycem*[tiab] OR hyper-glycaem*[tiab] OR "glucose flexibility"[tiab] OR "glucose flexibilities"[tiab] OR "glycemic flexibility"[tiab] OR "glycemic flexibilities"[tiab] OR "glycaemic flexibility"[tiab] OR "glycaemic flexibilities"[tiab] OR ((glucose[tiab] OR glycemic[tiab] OR glycaemic[tiab] OR glycemia[tiab] OR glycaemia[tiab]) AND (flexibilit*[tiab] OR deviation*[tiab])) OR glucose fluctuation*[tiab] OR glycemic fluctuation*[tiab] OR glycaemic fluctuation*[tiab] OR ((glycemia[tiab] OR glycaemia[tiab]) AND (fluctuation*[tiab])) OR glucose instabilit*[tiab] OR glycemic instabilit*[tiab] OR glycaemic instabilit*[tiab] OR ((glycemia[tiab] OR glycaemia[tiab]) AND (instabilit*[tiab])) OR glucose dynamic*[tiab] OR glycemic dynamic*[tiab] OR "glycaemic dynamics"[tiab] OR ((glycemia[tiab] OR glycaemia[tiab]) AND (dynamic*[tiab])) OR glucose stabilit*[tiab] OR glycemic stabilit*[tiab] OR glycaemic stabilit*[tiab] OR ((glycemia[tiab] OR glycaemia[tiab]) AND (stabilit*[tiab])) OR glucose excursion*[tiab] OR glycemic excursion*[tiab] OR glycaemic excursion*[tiab] OR ((glucose[tiab] OR glycemic[tiab] OR glycaemic[tiab] OR glycemia[tiab] OR glycaemia[tiab]) AND (stable[tiab] OR unstable[tiab])) | 115,865 |

**Embase.com Session Results (29 Jun 2018)**

| Search | Query | Items found |
| --- | --- | --- |
| #6 | #5 NOT ([animals]/lim NOT [humans]/lim) | 1,621 |
| #5 | #4 NOT (('adolescent'/exp OR 'child'/exp) NOT ('adult'/exp)) | 1,702 |
| #4 | #1 AND #2 AND #3 | 1,942 |
| #3 | 'psychology'/exp OR 'emotion'/exp/mj OR 'wellbeing'/exp OR emotion*:ab,ti,kw OR affect:ti OR affect:kw OR mood*:ab,ti,kw OR feelings:ab,ti,kw OR 'well-being':ab,ti,kw OR wellbeing:ab,ti,kw OR 'depressive symptom*':ab,ti,kw | 852,900 |
| #2 | 'diabetes mellitus'/exp OR diabetes:ab,ti,kw OR diabetic*:ab,ti,kw OR dm2:ab,ti,kw OR niddm:ab,ti,kw OR 'dm 2':ab,ti,kw OR t2d*:ab,ti,kw OR 'dm type 2':ab,ti,kw OR 'dm type II':ab,ti,kw OR dm1:ab,ti,kw OR iddm:ab,ti,kw OR 'dm 1':ab,ti,kw OR t1d*:ab,ti,kw OR 'dm type 1':ab,ti,kw OR 'dm type I':ab,ti,kw OR mody:ab,ti,kw OR lada:ab,ti,kw OR midd:ab,ti,kw | 1,009,306 |
| #1 | 'blood glucose':ab,ti,kw AND mood*:ab,ti,kw OR 'glucose rate of change*':ab,ti,kw OR 'blood sugar varia*':ab,ti,kw OR 'glucose varia*':ab,ti,kw OR 'glycemic varia*':ab,ti,kw OR 'glycaemic varia*':ab,ti,kw OR (((glycemia OR glycaemia) NEAR/3 varia*):ab,ti,kw) OR hypoglycem*:ab,ti,kw OR hypoglycaem*:ab,ti,kw OR 'hypo-glycem*':ab,ti,kw OR 'hypo-glycaem*':ab,ti,kw OR hyperglycem*:ab,ti,kw OR hyperglycaem*:ab,ti,kw OR 'hyper-glycem*':ab,ti,kw OR 'hyper-glycaem*':ab,ti,kw OR 'glucose flexibility':ab,ti,kw OR 'glucose flexibilities':ab,ti,kw OR 'glycemic flexibility':ab,ti,kw OR 'glycemic flexibilities':ab,ti,kw OR 'glycaemic flexibility':ab,ti,kw OR 'glycaemic flexibilities':ab,ti,kw OR (((glucose OR glycemic OR glycaemic OR glycemia OR glycaemia) NEAR/3 (flexibilit* OR deviation*)):ab,ti,kw) OR 'glucose fluctuation*':ab,ti,kw OR 'glycemic fluctuation*':ab,ti,kw OR 'glycaemic fluctuation*':ab,ti,kw OR (((glycemia OR glycaemia) NEAR/3 fluctuation*):ab,ti,kw) OR 'glucose instabilit*':ab,ti,kw OR 'glycemic instabilit*':ab,ti,kw OR 'glycaemic instabilit*':ab,ti,kw OR (((glycemia OR glycaemia) NEAR/3 instabilit*):ab,ti,kw) OR 'glucose dynamic*':ab,ti,kw OR 'glycemic dynamic*':ab,ti,kw OR 'glycaemic dynamics':ab,ti,kw OR (((glycemia OR glycaemia) NEAR/3 dynamic*):ab,ti,kw) OR 'glucose stabilit*':ab,ti,kw OR 'glycemic stabilit*':ab,ti,kw OR 'glycaemic stabilit*':ab,ti,kw OR glycemia:ab,ti,kw OR ((glycaemia NEAR/3 stabilit*):ab,ti,kw) OR 'glucose excursion*':ab,ti,kw OR 'glycemic excursion*':ab,ti,kw OR 'glycaemic excursion*':ab,ti,kw OR (((glucose OR glycemic OR glycaemic OR glycemia OR glycaemia) NEAR/3 (stable OR unstable)):ab,ti,kw) | 159,393 |

**Ebsco / PsycINFO Session Results (29 Jun 2018)**

| Search | Query | Results |
| --- | --- | --- |
| S5 | S4 NOT (PO Animal NOT PO Human) | 365 |
| S4 | S1 AND S2 AND S3 | 377 |
| S3 | DE "Psychology" OR DE "Emotions" OR DE "Emotional States" OR DE "Well Being" OR TI (emotion* OR affect OR mood* OR feelings OR "well-being" OR wellbeing OR "depressive symptom*") OR AB (emotion* OR  mood* OR feelings OR "well-being" OR wellbeing OR "depressive symptom*") | 558,459 |
| S2 | DE "Diabetes Mellitus" OR DE "Gestational Diabetes" OR DE "Type 2 Diabetes" OR DE "Blood Sugar" OR TI (diabetes OR diabetic* OR dm2 OR niddm OR "dm 2" OR t2d* OR "dm type 2" OR "dm type II" OR dm1 OR iddm OR "dm 1" OR t1d* OR "dm type 1" OR "dm type I" OR mody OR lada OR midd) OR AB (diabetes OR diabetic* OR dm2 OR niddm OR "dm 2" OR t2d* OR "dm type 2" OR "dm type II" OR dm1 OR iddm OR "dm 1" OR t1d* OR "dm type 1" OR "dm type I" OR mody OR lada OR midd) | 28,985 |
| S1 | TI ("blood glucose" AND mood*) OR "glucose rate of change*" OR "blood sugar varia*" OR "glucose varia*" OR "glycemic varia*" OR "glycaemic varia*" OR ((glycemia OR glycaemia) AND (varia*)) OR hypoglycem* OR hypoglycaem* OR "hypo-glycem*" OR "hypo-glycaem*" OR hyperglycem* OR hyperglycaem* OR "hyper-glycem*" OR "hyper-glycaem*" OR "glucose flexibility" OR "glucose flexibilities" OR "glycemic flexibility" OR "glycemic flexibilities" OR "glycaemic flexibility" OR "glycaemic flexibilities" OR ((glucose OR glycemic OR glycaemic OR glycemia OR glycaemia) AND (flexibilit* OR deviation*)) OR "glucose fluctuation*" OR "glycemic fluctuation*" OR "glycaemic fluctuation*" OR ((glycemia OR glycaemia) AND (fluctuation*)) OR "glucose instabilit*" OR "glycemic instabilit*" OR "glycaemic instabilit*" OR ((glycemia OR glycaemia) AND (instabilit*)) OR "glucose dynamic*" OR "glycemic dynamic*" OR "glycaemic dynamics" OR ((glycemia OR glycaemia) AND (dynamic*)) OR "glucose stabilit*" OR "glycemic stabilit*" OR "glycaemic stabilit*" OR ((glycemia OR glycaemia) AND (stabilit*)) OR "glucose excursion*" OR "glycemic excursion*" OR "glycaemic excursion*" OR ((glucose OR glycemic OR glycaemic OR glycemia OR glycaemia) AND (stable OR unstable)) OR AB ("blood glucose" AND mood*) OR "glucose rate of change*" OR "blood sugar varia*" OR "glucose varia*" OR "glycemic varia*" OR "glycaemic varia*" OR ((glycemia OR glycaemia) AND (varia*)) OR hypoglycem* OR hypoglycaem* OR "hypo-glycem*" OR "hypo-glycaem*" OR hyperglycem* OR hyperglycaem* OR "hyper-glycem*" OR "hyper-glycaem*" OR "glucose flexibility" OR "glucose flexibilities" OR "glycemic flexibility" OR "glycemic flexibilities" OR "glycaemic flexibility" OR "glycaemic flexibilities" OR ((glucose OR glycemic OR glycaemic OR glycemia OR glycaemia) AND (flexibilit* OR deviation*)) OR "glucose fluctuation*" OR "glycemic fluctuation*" OR "glycaemic fluctuation*" OR ((glycemia OR glycaemia) AND (fluctuation*)) OR "glucose instabilit*" OR "glycemic instabilit*" OR "glycaemic instabilit*" OR ((glycemia OR glycaemia) AND (instabilit*)) OR "glucose dynamic*" OR "glycemic dynamic*" OR "glycaemic dynamics" OR ((glycemia OR glycaemia) AND (dynamic*)) OR "glucose stabilit*" OR "glycemic stabilit*" OR "glycaemic stabilit*" OR ((glycemia OR glycaemia) AND (stabilit*)) OR "glucose excursion*" OR "glycemic excursion*" OR "glycaemic excursion*" OR ((glucose OR glycemic OR glycaemic OR glycemia OR glycaemia) AND (stable OR unstable)) | 5,264 |

## Search terms update May 2019

PubMed Session Results (26 May 2019)

| Search | Query | Items found |
| --- | --- | --- |
| #6 | #5 NOT ("Animals"[Mesh] NOT "Humans"[Mesh]) | 944 |
| #5 | #4 NOT (("Adolescent"[Mesh] OR "Child"[Mesh] OR "Infant"[Mesh]) NOT ("Adult"[Mesh])) | 998 |
| #4 | #1 AND #2 AND #3 | 1,111 |
| #3 | "Psychology"[Mesh] OR "Emotions"[Mesh] OR emotion*[tiab] OR affect[ti] OR affect[ot] OR mood*[tiab] OR feelings[tiab] OR well-being[tiab] OR wellbeing[tiab] OR depressive symptom*[tiab] | 593,484 |
| #2 | "Diabetes Mellitus"[Mesh] OR diabetes[tiab] OR diabetic*[tiab] OR dm2[tiab] OR niddm[tiab] OR dm 2[tiab] OR t2d*[tiab] OR dm type 2[tiab] OR dm type II[tiab] OR dm1[tiab] OR iddm[tiab] OR dm 1[tiab] OR t1d*[tiab] OR dm type 1[tiab] OR dm type I[tiab] OR mody[tiab] OR lada[tiab] OR midd[tiab] | 647,497 |
| #1 | (blood glucose[tiab] AND mood*[tiab]) OR glucose rate of change*[tiab] OR blood sugar varia*[tiab] OR glucose varia*[tiab] OR glycemic varia*[tiab] OR glycaemic varia*[tiab] OR ((glycemia[tiab] OR glycaemia[tiab]) AND (varia*[tiab])) OR hypoglycem*[tiab] OR hypoglycaem*[tiab] OR hypo-glycem*[tiab] OR hypo-glycaem*[tiab] OR hyperglycem*[tiab] OR hyperglycaem*[tiab] OR hyper-glycem*[tiab] OR hyper-glycaem*[tiab] OR "glucose flexibility"[tiab] OR "glucose flexibilities"[tiab] OR "glycemic flexibility"[tiab] OR "glycemic flexibilities"[tiab] OR "glycaemic flexibility"[tiab] OR "glycaemic flexibilities"[tiab] OR ((glucose[tiab] OR glycemic[tiab] OR glycaemic[tiab] OR glycemia[tiab] OR glycaemia[tiab]) AND (flexibilit*[tiab] OR deviation*[tiab])) OR glucose fluctuation*[tiab] OR glycemic fluctuation*[tiab] OR glycaemic fluctuation*[tiab] OR ((glycemia[tiab] OR glycaemia[tiab]) AND (fluctuation*[tiab])) OR glucose instabilit*[tiab] OR glycemic instabilit*[tiab] OR glycaemic instabilit*[tiab] OR ((glycemia[tiab] OR glycaemia[tiab]) AND (instabilit*[tiab])) OR glucose dynamic*[tiab] OR glycemic dynamic*[tiab] OR "glycaemic dynamics"[tiab] OR ((glycemia[tiab] OR glycaemia[tiab]) AND (dynamic*[tiab])) OR glucose stabilit*[tiab] OR glycemic stabilit*[tiab] OR glycaemic stabilit*[tiab] OR ((glycemia[tiab] OR glycaemia[tiab]) AND (stabilit*[tiab])) OR glucose excursion*[tiab] OR glycemic excursion*[tiab] OR glycaemic excursion*[tiab] OR ((glucose[tiab] OR glycemic[tiab] OR glycaemic[tiab] OR glycemia[tiab] OR glycaemia[tiab]) AND (stable[tiab] OR unstable[tiab])) | 122,206 |

**Embase.com Session Results (26 May 2019)**

| Search | Query | Items found |
| --- | --- | --- |
| #6 | #5 NOT ([animals]/lim NOT [humans]/lim) | 1,786 |
| #5 | #4 NOT (('adolescent'/exp OR 'child'/exp) NOT ('adult'/exp)) | 1,876 |
| #4 | #1 AND #2 AND #3 | 2,146 |
| #3 | 'psychology'/exp OR 'emotion'/exp/mj OR 'wellbeing'/exp OR emotion*:ab,ti,kw OR affect:ti OR affect:kw OR mood*:ab,ti,kw OR feelings:ab,ti,kw OR 'well-being':ab,ti,kw OR wellbeing:ab,ti,kw OR 'depressive symptom*':ab,ti,kw | 931,140 |
| #2 | 'diabetes mellitus'/exp OR diabetes:ab,ti,kw OR diabetic*:ab,ti,kw OR dm2:ab,ti,kw OR niddm:ab,ti,kw OR 'dm 2':ab,ti,kw OR t2d*:ab,ti,kw OR 'dm type 2':ab,ti,kw OR 'dm type II':ab,ti,kw OR dm1:ab,ti,kw OR iddm:ab,ti,kw OR 'dm 1':ab,ti,kw OR t1d*:ab,ti,kw OR 'dm type 1':ab,ti,kw OR 'dm type I':ab,ti,kw OR mody:ab,ti,kw OR lada:ab,ti,kw OR midd:ab,ti,kw | 1,081,849 |
| #1 | ('blood glucose':ab,ti,kw AND mood*:ab,ti,kw) OR 'glucose rate of change*':ab,ti,kw OR 'blood sugar varia*':ab,ti,kw OR 'glucose varia*':ab,ti,kw OR 'glycemic varia*':ab,ti,kw OR 'glycaemic varia*':ab,ti,kw OR (((glycemia OR glycaemia) NEAR/3 varia*):ab,ti,kw) OR hypoglycem*:ab,ti,kw OR hypoglycaem*:ab,ti,kw OR 'hypo glycem*':ab,ti,kw OR 'hypo glycaem*':ab,ti,kw OR hyperglycem*:ab,ti,kw OR hyperglycaem*:ab,ti,kw OR 'hyper glycem*':ab,ti,kw OR 'hyper glycaem*':ab,ti,kw OR 'glucose flexibility':ab,ti,kw OR 'glucose flexibilities':ab,ti,kw OR 'glycemic flexibility':ab,ti,kw OR 'glycemic flexibilities':ab,ti,kw OR 'glycaemic flexibility':ab,ti,kw OR 'glycaemic flexibilities':ab,ti,kw OR (((glucose OR glycemic OR glycaemic OR glycemia OR glycaemia) NEAR/3 (flexibilit* OR deviation*)):ab,ti,kw) OR 'glucose fluctuation*':ab,ti,kw OR 'glycemic fluctuation*':ab,ti,kw OR 'glycaemic fluctuation*':ab,ti,kw OR (((glycemia OR glycaemia) NEAR/3 fluctuation*):ab,ti,kw) OR 'glucose instabilit*':ab,ti,kw OR 'glycemic instabilit*':ab,ti,kw OR 'glycaemic instabilit*':ab,ti,kw OR (((glycemia OR glycaemia) NEAR/3 instabilit*):ab,ti,kw) OR 'glucose dynamic*':ab,ti,kw OR 'glycemic dynamic*':ab,ti,kw OR 'glycaemic dynamics':ab,ti,kw OR (((glycemia OR glycaemia) NEAR/3 dynamic*):ab,ti,kw) OR 'glucose stabilit*':ab,ti,kw OR 'glycemic stabilit*':ab,ti,kw OR 'glycaemic stabilit*':ab,ti,kw OR glycemia:ab,ti,kw OR ((glycaemia NEAR/3 stabilit*):ab,ti,kw) OR 'glucose excursion*':ab,ti,kw OR 'glycemic excursion*':ab,ti,kw OR 'glycaemic excursion*':ab,ti,kw OR (((glucose OR glycemic OR glycaemic OR glycemia OR glycaemia) NEAR/3 (stable OR unstable)):ab,ti,kw) | 170,552 |

**Ebsco / PsycINFO Session Results (26 May 2019)**

| Search | Query | Items found |
| --- | --- | --- |
| S5 | S4 NOT (PO Animal NOT PO Human) | 319 |
| S4 | S1 AND S2 AND S3 | 329 |
| S3 | DE "Psychology" OR DE "Emotions" OR DE "Emotional States" OR DE "Well Being" OR TI (emotion* OR affect OR mood* OR feelings OR "well-being" OR wellbeing OR "depressive symptom*") OR AB (emotion* OR  mood* OR feelings OR "well-being" OR wellbeing OR "depressive symptom*") OR KW (emotion* OR  mood* OR feelings OR "well-being" OR wellbeing OR "depressive symptom*") | 595,914 |
| S2 | DE "Diabetes Mellitus" OR DE "Gestational Diabetes" OR DE "Type 2 Diabetes" OR DE "Blood Sugar" OR TI (diabetes OR diabetic* OR dm2 OR niddm OR "dm 2" OR t2d* OR "dm type 2" OR "dm type II" OR dm1 OR iddm OR "dm 1" OR t1d* OR "dm type 1" OR "dm type I" OR mody OR lada OR midd) OR AB (diabetes OR diabetic* OR dm2 OR niddm OR "dm 2" OR t2d* OR "dm type 2" OR "dm type II" OR dm1 OR iddm OR "dm 1" OR t1d* OR "dm type 1" OR "dm type I" OR mody OR lada OR midd) OR KW (diabetes OR diabetic* OR dm2 OR niddm OR "dm 2" OR t2d* OR "dm type 2" OR "dm type II" OR dm1 OR iddm OR "dm 1" OR t1d* OR "dm type 1" OR "dm type I" OR mody OR lada OR midd) | 30,514 |
| S1 | TI (("blood glucose" AND mood*) OR "glucose rate of change*" OR "blood sugar varia*" OR "glucose varia*" OR "glycemic varia*" OR "glycaemic varia*" OR ((glycemia OR glycaemia) AND (varia*)) OR hypoglycem* OR hypoglycaem* OR "hypo-glycem*" OR "hypo-glycaem*" OR hyperglycem* OR hyperglycaem* OR "hyper-glycem*" OR "hyper-glycaem*" OR "glucose flexibility" OR "glucose flexibilities" OR "glycemic flexibility" OR "glycemic flexibilities" OR "glycaemic flexibility" OR "glycaemic flexibilities" OR ((glucose OR glycemic OR glycaemic OR glycemia OR glycaemia) AND (flexibilit* OR deviation*)) OR "glucose fluctuation*" OR "glycemic fluctuation*" OR "glycaemic fluctuation*" OR ((glycemia OR glycaemia) AND (fluctuation*)) OR "glucose instabilit*" OR "glycemic instabilit*" OR "glycaemic instabilit*" OR ((glycemia OR glycaemia) AND (instabilit*)) OR "glucose dynamic*" OR "glycemic dynamic*" OR "glycaemic dynamics" OR ((glycemia OR glycaemia) AND (dynamic*)) OR "glucose stabilit*" OR "glycemic stabilit*" OR "glycaemic stabilit*" OR ((glycemia OR glycaemia) AND (stabilit*)) OR "glucose excursion*" OR "glycemic excursion*" OR "glycaemic excursion*" OR ((glucose OR glycemic OR glycaemic OR glycemia OR glycaemia) AND (stable OR unstable))) OR AB (("blood glucose" AND mood*) OR "glucose rate of change*" OR "blood sugar varia*" OR "glucose varia*" OR "glycemic varia*" OR "glycaemic varia*" OR ((glycemia OR glycaemia) AND (varia*)) OR hypoglycem* OR hypoglycaem* OR "hypo-glycem*" OR "hypo-glycaem*" OR hyperglycem* OR hyperglycaem* OR "hyper-glycem*" OR "hyper-glycaem*" OR "glucose flexibility" OR "glucose flexibilities" OR "glycemic flexibility" OR "glycemic flexibilities" OR "glycaemic flexibility" OR "glycaemic flexibilities" OR ((glucose OR glycemic OR glycaemic OR glycemia OR glycaemia) AND (flexibilit* OR deviation*)) OR "glucose fluctuation*" OR "glycemic fluctuation*" OR "glycaemic fluctuation*" OR ((glycemia OR glycaemia) AND (fluctuation*)) OR "glucose instabilit*" OR "glycemic instabilit*" OR "glycaemic instabilit*" OR ((glycemia OR glycaemia) AND (instabilit*)) OR "glucose dynamic*" OR "glycemic dynamic*" OR "glycaemic dynamics" OR ((glycemia OR glycaemia) AND (dynamic*)) OR "glucose stabilit*" OR "glycemic stabilit*" OR "glycaemic stabilit*" OR ((glycemia OR glycaemia) AND (stabilit*)) OR "glucose excursion*" OR "glycemic excursion*" OR "glycaemic excursion*" OR ((glucose OR glycemic OR glycaemic OR glycemia OR glycaemia) AND (stable OR unstable))) OR KW (("blood glucose" AND mood*) OR "glucose rate of change*" OR "blood sugar varia*" OR "glucose varia*" OR "glycemic varia*" OR "glycaemic varia*" OR ((glycemia OR glycaemia) AND (varia*)) OR hypoglycem* OR hypoglycaem* OR "hypo-glycem*" OR "hypo-glycaem*" OR hyperglycem* OR hyperglycaem* OR "hyper-glycem*" OR "hyper-glycaem*" OR "glucose flexibility" OR "glucose flexibilities" OR "glycemic flexibility" OR "glycemic flexibilities" OR "glycaemic flexibility" OR "glycaemic flexibilities" OR ((glucose OR glycemic OR glycaemic OR glycemia OR glycaemia) AND (flexibilit* OR deviation*)) OR "glucose fluctuation*" OR "glycemic fluctuation*" OR "glycaemic fluctuation*" OR ((glycemia OR glycaemia) AND (fluctuation*)) OR "glucose instabilit*" OR "glycemic instabilit*" OR "glycaemic instabilit*" OR ((glycemia OR glycaemia) AND (instabilit*)) OR "glucose dynamic*" OR "glycemic dynamic*" OR "glycaemic dynamics" OR ((glycemia OR glycaemia) AND (dynamic*)) OR "glucose stabilit*" OR "glycemic stabilit*" OR "glycaemic stabilit*" OR ((glycemia OR glycaemia) AND (stabilit*)) OR "glucose excursion*" OR "glycemic excursion*" OR "glycaemic excursion*" OR ((glucose OR glycemic OR glycaemic OR glycemia OR glycaemia) AND (stable OR unstable))) | 4,318 |

# Supplementary File 2: Inclusion and exclusion criteria full text selection

| Inclusion criteria | Exclusion criteria |
| --- | --- |
| An article/study that:  Involves (one of the) parameters of glucose variability:   - Glucose- / Glyce(a)mic-/Blood sugar-… - Variability - Variation - Flexibility/Flexibilities - Fluctuation - Instability/ Instabilities - Dynamic(s) - Change(s) - Excursions - Stability - (Un)stable)   Involves (one of the) aspects of mood:   - Emotion - Affect - Mood - Feelings - Wellbeing - Depressive symptoms - Anxiety   Analyzed and described the association between glucose variability and psychological wellbeing, included in a statistical analysis as (in)dependent variables.  Was conducted in adults, with (all types of) diabetes mellitus  Study design: observational, experimental. | An article/study that:  Was conducted in children or adolescents or parents/partners or animals  Glucose *only* measured in one of the following ways:   - HbA1c - Fasting glucose   Study design: case reports and qualitative studies. |

# Supplementary File 3: Quality assessment

Based on National Institutes of Health (NIH) study quality assessment tools for Observational Cohort and Cross-Sectional Studies.

|  | Ahola 2018 ^1^ | Cox 2007 ^2^ | Gonder-Frederick 1990 ^3^ | Hermanns  2007 ^4^ | Johansson 1999 ^5^ | Kovachev 2003 ^6^ | Penckofer 2012 ^7^ | Wagner 2017 ^8^ |
| --- | --- | --- | --- | --- | --- | --- | --- | --- |
| 1. Was the research question or objective in this paper clearly stated? | Yes | Yes | Yes | Yes | Yes | Yes | No | Yes |
| 1. Was the study population clearly specified and defined? | No | No | No | Yes | No | No | No | Yes |
| 1. Was the participation rate of eligible persons at least 50%? | NR | Yes | NR | CD | NR | NR | NR | Yes |
| 1. Were all the subjects selected or recruited from the same or similar populations (including the same time period)? Were inclusion and exclusion criteria for being in the study prespecified and applied uniformly to all participants? | NR | NR | No | NR | NR | NR | NR | Yes |
| 1. Was a sample size justification, power description, or variance and effect estimates provided? | No | No | No | No | Yes | NR | No | No |
| 1. For the analyses of the relationship between GV and mood, were the exposure(s) of interest measured prior to the outcome(s) being measured? | Yes | Yes | Yes | Yes | Yes | Yes | Yes | Yes |
| 1. Was the timeframe sufficient so that one could reasonably expect to see an association between exposure and outcome if it existed to answer research question? | Yes | Yes | Yes | Yes | Yes | Yes | Yes | Yes |
| 1. For exposures that can vary in amount or level, did the study examine different levels of the exposure as related to the outcome (e.g., categories of exposure, or exposure measured as continuous variable)? | Yes | Yes | Yes | Yes | Yes | Yes | Yes | Yes |
| 1. Were the exposure measures (independent variables) clearly defined, valid, reliable, and implemented consistently across all study participants? | Yes | Yes | Yes | Yes | Yes | Yes | Yes | Yes |
| 1. Was the exposure(s) assessed more than once over time? | Yes | Yes | Yes | Yes | Yes | Yes | Yes | Yes |
| 1. Were the outcome measures (dependent variables) clearly defined, valid, reliable, and implemented consistently across all study participants? | Yes | Yes | Yes | Yes | Yes | Yes | Yes | Yes |
| 1. Were the outcome assessors blinded to the exposure status of participants? | NA | NA | No | Yes | NR | NA | NA | NA |
| 1. Was loss to follow-up after baseline 20% or less? | NA | Yes | Yes | NR | Yes | Yes | No | Yes |
| 1. Were key potential confounding variables measured and adjusted statistically for their impact on the relationship between exposure(s) and outcome(s)? | Yes | No | Yes | No | No | No | Yes | No |
| **Total score: Number of Yes** | 8 | 9 | 9 | 9 | 9 | 8 | 7 | 11 |
| **Overall quality rating** | Fair | Fair | Fair | Fair | Fair | Fair | Poor | Fair |

* CD: cannot determine; NR: Not reported; NA: Not applicable

1. Ahola AJ, Forsblom C, Groop PH. Association between depressive symptoms and dietary intake in patients with type 1 diabetes. *Diabetes Res Clin Pract.* 2018;139:91-99.

2. Cox DJ, McCall A, Kovatchev B, Sarwat S, Ilag LL, Tan MH. Effects of blood glucose rate of changes on perceived mood and cognitive symptoms in insulin-treated type 2 diabetes. *Diabetes Care.* 2007;30(8):2001-2002.

3. Gonder-Frederick LA, Carter WR, Cox DJ, Clarke WL. Environmental stress and blood glucose change in insulin-dependent diabetes mellitus. *Health Psychol.* 1990;9(5):503-515.

4. Hermanns N, Scheff C, Kulzer B, et al. Association of glucose levels and glucose variability with mood in type 1 diabetic patients. *Diabetologia.* 2007;50(5):930-933.

5. Johansson UB, Wredling RA, Adamson UC, Lins PE. A randomised study evaluating the effects of cisapride on glucose variability and quality of life parameters in insulin-dependent diabetes mellitus patients. *Diabetes Metab.* 1999;25(4):314-319.

6. Kovatchev B, Cox DJ, Summers KH, Gonder-Frederick L, Clarke WL. Postprandial glucose dynamics and associated symptoms in type 2 diabetes mellitus. *J Appl Res.* 2003;3(4):449-458.

7. Penckofer S, Quinn L, Byrn M, Ferrans C, Miller M, Strange P. Does glycemic variability impact mood and quality of life? *Diabetes Technol Ther.* 2012;14(4):303-310.

8. Wagner J, Armeli S, Tennen H, Bermudez-Millan A, Wolpert H, Perez-Escamilla R. Mean Levels and Variability in Affect, Diabetes Self-Care Behaviors, and Continuously Monitored Glucose: A Daily Study of Latinos With Type 2 Diabetes. *Psychosom Med.* 2017;79(7):798-805.
